# Hemoptysis Secondary to Anomalous Origin of Right Pulmonary Artery From Ascending Aorta in a Young Male: A Case Report

**DOI:** 10.7759/cureus.51634

**Published:** 2024-01-04

**Authors:** Simran Nimal, Gowrishankar Palaniswamy, Navya Pillikunte Doddareddy, Sahithi Talacheru, Shraddha Jadhav, Tanmayee Mareedu, Mihirkumar P Parmar, Anup Banur

**Affiliations:** 1 Internal Medicine, Byramjee Jeejeebhoy Government Medical College, Pune, IND; 2 Internal Medicine, Saveetha Medical College and Hospital, Chennai, IND; 3 Internal Medicine, Bangalore Medical College and Research Institute, Bangalore, IND; 4 Internal Medicine, MediCiti Institute of Medical Sciences, Hyderabad, IND; 5 Internal Medicine, Teaching University Geomedi, Tbilisi, GEO; 6 Internal Medicine, Mamata Academy of Medical Sciences, Hyderabad, IND; 7 Internal Medicine, Gujarat Medical Education and Research Society, Vadodara, IND; 8 Pulmonology, S. S. Institute of Medical Sciences and Research Centre, Davanagere, IND

**Keywords:** congenital, dyspnea, hemoptysis, anomalous origin, pda

## Abstract

We report a rare case of a 24-year-old male with a rare anatomic variant of patent ductus arteriosus (PDA). The patient presented with symptoms of productive cough with recurrent and severe bouts of hemoptysis and grade I dyspnea. There were no prior episodes reported. The patient was vitally stable with bilateral clubbing. On cardiopulmonary auscultation, a prominent parasternal heave, loud P2, and right lung crepitus were noted. A complete blood count revealed an elevated hemoglobin and RBC count. An ECG revealed sinus tachycardia and right ventricle (RV) strain. ECHO confirmed these findings, as dilated right atrium (RA) and RV, mild tricuspid valve regurgitation (TR), and severe pulmonary hypertension were noted. CT of the chest demonstrated multiple ground glass opacities, right lung consolidation, and volume loss suggestive of right-sided pneumonia with atelectasis. CT also proved the presence of PDA and an anomalous origin of the right pulmonary artery from the right ascending aorta, causing compression of the right main bronchus. We show the clinical and radiological findings and discuss the implications and approach to this rare congenital cardiovascular malformation, as well as how a patient-centered approach is necessary for its management.

## Introduction

This article was previously posted to the Authorea preprint server on November 18, 2023 [1].

Adults with congenital heart disease (CHD) represent an expanding patient population requiring life-long tertiary medical care. Approximately 5% to 10% of them develop pulmonary arterial hypertension (PAH) of variable severity [2]. The abnormal origin of the pulmonary artery means that the pathological branch comes from the ascending aorta. This happens about 0.12% of the time [3]; thus, it's often a case of misdiagnosis or missed diagnosis. In the presence of an additional shunt-like anomalous origin of the pulmonary artery (AOPA), left-to-right shunting caused by patent ductus arteriosus (PDA) becomes more pronounced. AOPA, coupled with PDA, could present with lethal complications like hemoptysis in the clinical course of disabling pulmonary hypertension [4]. Hemoptysis occurs in approximately 3.1% to 5.5% of PAH-CHD patients, making it a rare clinical presentation [5]. Follow-up is key for these patients to promptly detect new or misdiagnosed pathologic findings [4]. Patients with CHD-PAH have a survival advantage compared to those with other types of PAH who present with hemoptysis, thus necessitating prompt recognition and multi-centered treatment strategies [6]. Numerous strategies employed in the past include supportive care, surgical resection, and lung transplants. Presently, the most commonly used strategy is bronchial artery embolization (BAE) [6]. BAE is minimally invasive and has proved to be an immense success, resulting in rapid cessation of hemoptysis with low complication rates [5]. Recent studies have shown the effectiveness of tranexamic acid in reducing the volume and duration of hemoptysis [7]. Even though there have been big steps forward in PAH-targeted treatment strategies and techniques, hemoptysis is still a major cause of death and disability, lowering people's quality of life and having an effect on healthcare systems around the world. This means that each case needs to be treated individually and with close collaboration between different fields of medicine. This includes collaboration among adult CHD experts, pulmonologists, radiologists, and thoracic surgeons in tertiary centers [4,5].

## Case presentation

A 24-year-old male patient presented to the Outpatient Department with chief complaints of cough with expectorations associated with recurrent and severe bouts of hemoptysis for five days. The patient also experienced Grade 1 dyspnea on the Modified Medical Research Council Dyspnea Scale (mMRC). The patient denied having any chest pain or palpitations. The patient had neither a fever nor any similar complaints in the past and also denied having a history of pulmonary tuberculosis contact. The COVID-19 rapid antigen test yielded a negative result. The patient also denied having a history of any bleeding disorder or anticoagulation use.

Physical examination

**Figure 1:**
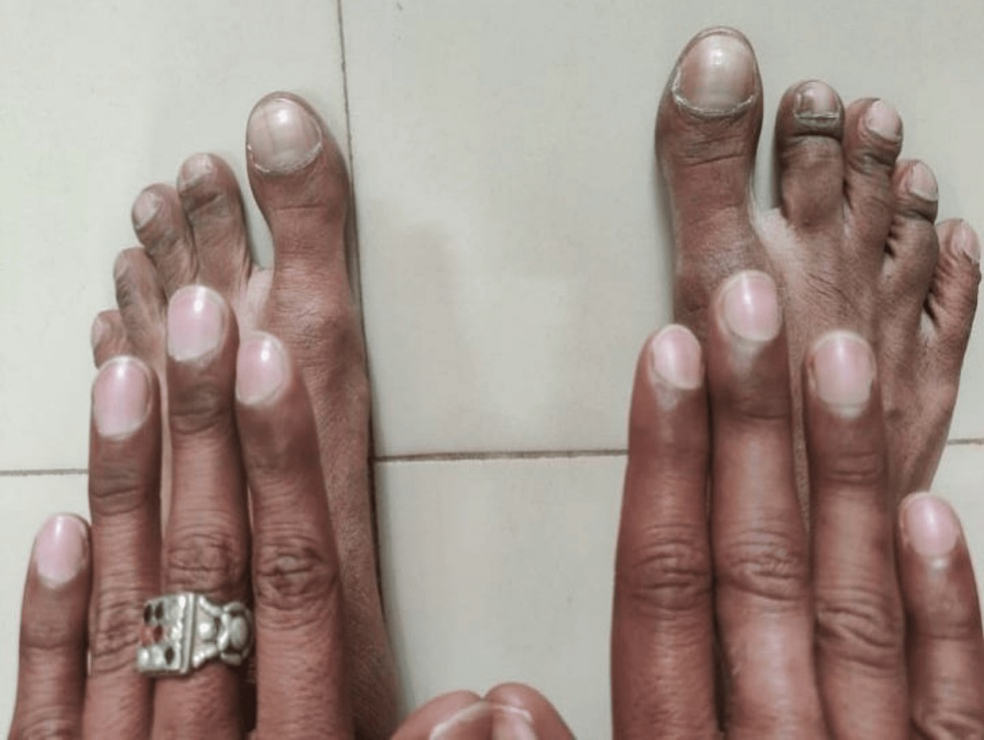
Bilateral pan-digital clubbing

On admission, the patient looked unwell but had normal vital signs (temperature, 36.3 C; blood pressure, 133/81 mmHg; pulse, 96 beats/minute; respiratory rate, 22 breaths/minute; oxygen saturation, Spo2: 97% room air). The patient had bilateral pan-digital clubbing (Figure 1). A cardio-pulmonary examination yielded findings such as a prominent parasternal heave. Chest wall auscultation revealed a loud P2 in the left parasternal space, as well as crepitus in the right lung fields.

Laboratory findings

Table 1 provides the results of the initial blood tests, which included a complete blood count and metabolic panel. The blood tests revealed elevated hemoglobin levels and an elevated RBC count in the patient.

**Table 1:** Results of complete blood count CBC: complete blood count, MCV: mean corpuscular volume, MCH: mean corpuscular hemoglobin, MCHC: mean corpuscular hemoglobin concentration, RDW-CV: red cell distribution width – coefficient of variation

Parameter	Result	Reference values
CBC		
1) Hemoglobin	20.3	13-18 g/dl
2) RBC count	7.8 million/cumm	4.5-6.5 million/cumm
3) Total count	7440 cells/cumm	4000-11,000 cells/cumm
Differential leukocyte count		
1) Neutrophils	67%	40-75%
2) Lymphocytes	25%	20-45%
3) Eosinophils	2%	1-6%
4) Monocytes	6%	2-10%
Packed Cell Volume (hematocrit)	63.2%	47-77%
MCV	81 fl	76-96 fl
MCH	26 pg	27-32 pg
MCHC	31.1%	30-35%
RDW-CV	19.4	12-15
Platelet count	1.30 lakhs/cumm	1.5-4.5 lakhs/cumm

Clinical course

**Figure 2:**
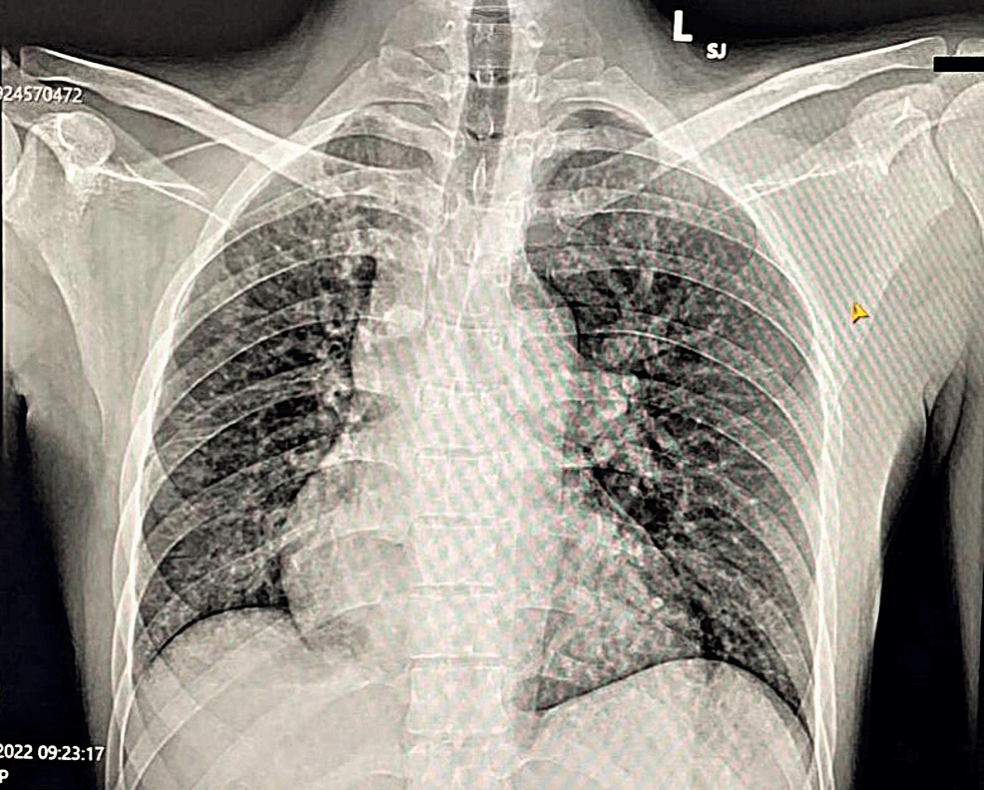
Chest X-ray, posteroanterior view Enlarged cardiac silhouette with prominent pulmonary trunk and pulmonary arteries proximally

This prompted further investigations. The chest X-ray showed a bigger heart shape with the pulmonary trunk and pulmonary arteries closer together (Figure 2). The ECG showed sinus tachycardia with right ventricular strain. An ECHO was done to confirm these findings; it revealed a dilated right atrium (RA), right ventricle (RV), and mild tricuspid valve regurgitation (TR). The most notable finding was severe pulmonary artery hypertension (62 mmHg).

**Figure 3:**
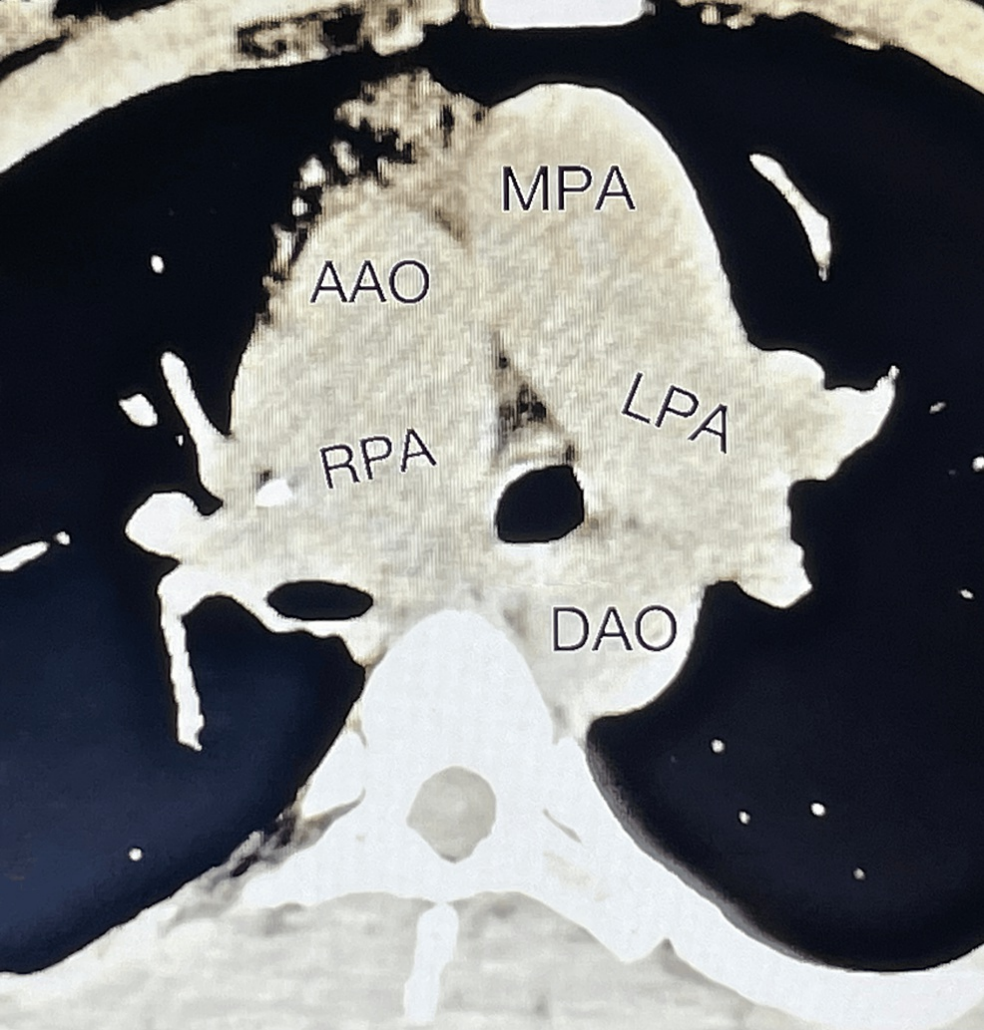
Chest CT scan PDA and an abnormal origin of the right pulmonary artery from the right ascending aorta. AAO: ascending aorta, RPA: right pulmonary artery, DAO: descending aorta, LPA: left pulmonary artery, LV: left ventricle, MPA: main pulmonary artery

Doctors performed a chest CT to determine the cause of the high blood pressure in the pulmonary arteries. The scan showed volume loss in the right hemithorax. The CT scan also showed a PDA and an abnormal origin of the right pulmonary artery from the right ascending aorta, which has compressed the right main bronchus (Figure 3). Due to ongoing hemoptysis and infection, we avoided performing a spirometry analysis.

Hemoptysis with atelectasis was the final diagnosis. This was due to the PDA and an abnormal origin of the right pulmonary artery from the ascending aorta, which pushed on the right main bronchus. We managed the hemoptysis conservatively. To manage his pneumonia, the medical team administered supplementary oxygen and IV antibiotics to the patient for three days.

However, the patient returned a year later, in 2022, with similar complaints. During this episode, the medical team again managed the patient conservatively with antifibrinolytics and cough suppressants and advised a 10-day rest period. However, the medical team added Tab. Tadalafil 20 mg is used to control pulmonary hypertension. To avoid any further infectious complications, the healthcare provider administered the patient the pneumococcal and annual flu vaccines. The healthcare provider advised the patient to schedule follow-up visits in three months to closely monitor and improve pulmonary hypertension.

## Discussion

Congenital heart defects like PDA cause left-to-right shunts. This shunt causes the pulmonary vasculature to undergo pathological changes such as hypertrophy and fibrosis, consequently causing PAH. Initially, most patients are asymptomatic until a significant rise in pulmonary arterial pressure occurs and is thus present in adulthood, as seen in this patient [8]. Exercise intolerance, palpitations, and arrhythmias are all different presentations of PAH. In this case [9], only 12% of adult survivors experience hemoptysis, which is a rare presentation. These presentations are more pronounced in the presence of an additional shunt. As noted in this patient, there is another shunt between the right pulmonary artery and the aorta. To the best of our knowledge, this is the first anatomic variant of PDA reported and thus warrants a very patient-centered approach concerning its management.

The workup for hemoptysis invariably depends on the past medical history, which in our case was not significant despite the excessive pulmonary hypertension (68 mmHg on echocardiography) seen in this patient and is a rather rare clinical picture [10]. Chest X-ray remains the best initial test of choice [11]. It determines the site of bleeding in 45 to 65% of the cases and the cause in 25% to 35% [12]. The next step has been a subject of debate as to whether bronchoscopy is better than CT in determining the cause of hemoptysis; however, recent studies demonstrate the superiority of CT [10]. In a case of active hemoptysis, such as ours, bronchoscopy can stimulate coughing and therefore increase the risk of bleeding. Therefore, it is often safer to postpone the bronchoscopy until a definitive diagnosis is made [10]. A radiologic definition based on CT states that pulmonary artery hypertension is diagnosed when the pulmonary artery diameter is more than 28 mm [12]. In our case, this is conclusively proven by a pulmonary artery diameter of 38 mm [13]. CT scans can help doctors evaluate people with pulmonary hypertension in a number of ways. They can give important information about the condition's possible causes, such as lung parenchymal disease or primary cardiac processes, as well as chronic thromboembolic disease. They can also clearly show the current condition of the RV, which is an important part of patient care in pulmonary hypertension cases. Doctors use echocardiography to assess the likelihood of PAH and determine survival outcomes based on its parameters [14].

Embolotherapy is a widely accepted treatment modality for patients with severe recurrent hemoptysis [9]. Embolotherapy has been proven to achieve immediate bleeding control in up to 90% of patients, but it carries a significant risk of re-bleeding in approximately 50% of patients during long-term follow-up [15]. Recent studies have shown that acute presentations, like in this case, if treated surgically, present with more complications in the long term [12]. Studies have shown that a surgical approach is also associated with high operative mortality (>15%) [9]. On the contrary, conservative management has shown better patient outcomes in some patients [12]. This includes a combination of cough suppressants, antifibrinolytics like tranexamic acid, and antibiotics. Studies have shown a significant reduction in the volume and duration of hemoptysis with the use of antifibrinolytic agents, thus reducing morbidity [7]. The other spectrum of management is to control and adequately monitor PAH, which is the major cause of mortality in patients with multiple left-to-right shunts.

Tadalafil effectively controls PAH when given once a day due to its high concentration of PDE5 in the lungs [16,17]. Furthermore, when compared to alternative treatment methods [18], tadalafil is regarded as the most economical choice. Hence, conclusively, we state that, due to the rare anatomic variant as well as the pronounced symptoms present in this patient, it might prove counter-intuitive to plan a surgical approach and have preferentially selected the aforementioned conservative approach.

## Conclusions

In instances of this nature, the individual is required to undergo a series of procedures and investigations. The evaluation process relies on the individual's medical history, which, in this instance, is not significant. The rarity of this case is attributed to the presence of significant pulmonary hypertension characterized by a pulmonary artery diameter measuring 38 mm and the additional active hemoptysis, which necessitated a delay in performing bronchoscopy due to the potential risk of bleeding. Despite being a well-acknowledged therapeutic technique for patients experiencing severe recurrent hemoptysis and providing prompt control of bleeding, embolotherapy still carries a notable risk of re-bleeding. When surgical intervention is employed, the heightened severity of the illness, along with the patient's atypical anatomical variation of the PDA in relation to the aorta and the severity of the symptoms presented, may lead to exacerbated long-term complications. Furthermore, this particular instance serves as a cautionary tale for medical professionals to exercise vigilant monitoring of patients presenting with identical symptoms and displaying signs of pulmonary hypertension.
